# Bioactive Peptides from Milk Proteins with Antioxidant, Anti-Inflammatory, and Antihypertensive Activities

**DOI:** 10.3390/foods14030535

**Published:** 2025-02-06

**Authors:** Thaís Borges, Pedro Coelho, Cristina Prudêncio, Ana Gomes, Paula Gomes, Ricardo Ferraz

**Affiliations:** 1 LAQV-REQUIMTE, Department of Chemistry and Biochemistry, Faculty of Sciences, University of Porto, Rua do Campo Alegre, S/N, 4169-007 Porto, Portugal; thaiscgborges@gmail.com (T.B.); agomes@fc.up.pt (A.G.); pgomes@fc.up.pt (P.G.); 2 RISE-Health, Center for Translational Health and Medical Biotechnology Research (TBIO), ESS, Polytechnic of Porto, R. Dr. António Bernardino de Almeida, 400, 4200-072 Porto, Portugal; pvc@ess.ipp.pt (P.C.); cprudencio@ess.ipp.pt (C.P.); 3 Chemical and Biomolecular Sciences, School of Health (ESS), Polytechnic Institute of Porto, 4200-072 Porto, Portugal

**Keywords:** bioactive peptides, milk proteins, antioxidant, anti-inflammatory, antihypertensive

## Abstract

Background/Objectives: Peptides from protein ingredients exhibit key biological activities, including antimicrobial, antihypertensive, antioxidant, anti-inflammatory, analgesic, and immunomodulatory effects. Aligning with the One Health approach, there is growing investment in promoting pet health and well-being. As a result, sustainable functional ingredients are increasingly essential for pet food development. In this work, peptides derived from lactoferrins of different mammalian species were synthesized and their antioxidant, anti-inflammatory, and antihypertensive activities were investigated. Methods: This study examined the antioxidant, anti-inflammatory, antihypertensive activities, and cytotoxicity of bioactive peptides derived from lactoferrins of various mammalian species through spectroscopical methods. The peptides were produced via chemical synthesis (bottom-up approach). Results: Peptides derived from bovine lactoferrin showed the most promising antioxidant and anti-inflammatory activities, whereas those derived from human lactoferrin showed the highest antihypertensive effects and the lowest cytotoxicity. In short, milk-derived peptides with antioxidant, anti-inflammatory, and antihypertensive activity were identified. Conclusions: This motivates further studies to better characterize these peptides, including their properties and pharmacokinetics in vivo, to assess their true potential as nutraceutical agents.

## 1. Introduction

Food-derived bioactive peptides (FBPs) are small sequences within food proteins that exert a positive impact on body function or condition [1,2]. Over recent decades, FBPs have been receiving a lot of attention, mainly due to their potential health advantages and also due to their diverse applications even beyond the food industry (e.g., in the pharmaceutical and cosmetic sectors) [3].

The identification and optimization of FBPs that could have a potential impact on health have expanded in recent years due to the development of several chemical and biological methods for their production and modification [1,4]. These methods encompass either top-down approaches, i.e., the isolation of the FBPs directly from their protein source [5], or bottom-up approaches in which FBPs with a pre-determined sequence are produced by chemical synthesis or recombinant technology [6]. All these tools enabled the gathering of a significant portfolio of FBPs and the development of databases such as the DFBP database freely available via http://www.cqudfbp.net/ (access on 3 February 2025) [7]. Among the multiple FBPs that have been identified thus far, those derived from milk have gained particular attention since quite a few years ago [8] and remain under the spotlight due to their large potential both as therapeutics [9,10] and as cosmetics [11,12,13]. This explains the existence of databases exclusively devoted to milk-derived bioactive peptides (MDBPs) [14,15] and their frequent updating [16]. Although the most widely explored MDBPs originate from bovine [17,18] and human [19,20,21] milk proteins, the search for bioactive components, including peptides, in milk products of minor dairy species, such as sheep, goat, camel, buffalo, donkey, and horse, is gaining momentum [22].

Inspired by the above and following our long-term interest in the antimicrobial properties of bioactive peptides derived from human (hLF) and bovine (bLF) lactoferrins [23,24,25]. This study investigates the potential health benefits of peptides derived from donkey (*Equus africanus asinus*) milk. The relevant peptides were produced through standard solid-phase peptide synthesis (SPPS) [26]. The focus was on assessing their antioxidant, anti-inflammatory, and antihypertensive effects. The novelty of this research lies in exploring these specific health benefits and evaluating the effects of these peptides in vitro and in cellulo.

## 2. Materials and Methods

### 2.1. Chemicals

All general-purpose reagents and solvents, in vitro assay kits, and disposable glassware/plasticware were purchased and used as received from the following suppliers: Sigma-Aldrich (St. Louis, MO, USA), Corning (New York, NY, USA), Alfa Aesar (Ward Hill, MA, USA), Fisher Chemicals (Waltham, MA, USA), TCI Chemicals (Tokyo, Japan), Randox Laboratories (Crumlin, UK), Abcam (Cambridgeshire, UK), and Chem-Lab (Zedelgem, Belgium). For SPPS, the Rink amide MBHA resin (100–200 mesh, 0.52 mmol/g) and the in situ coupling reagent O-(6-chlorobenzotriazol-1-yl)-N,N,N′,N′-tetramethyluronium hexafluorophosphate (HCTU) were from NovaBiochem (Merck Millipore, Burlington, MA, USA), the Fmoc-protected amino acids (Fmoc-AA-OH) were from Bachem (Bubendorf, Switzerland), and all other chemicals were from Sigma-Aldrich (St. Louis, MO, USA).

### 2.2. Peptide Synthesis

**Scheme 1:**
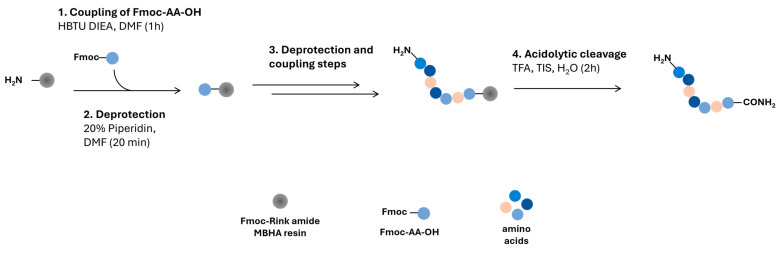
Schematic figure of the peptide synthesis procedure.

The peptides (Table 1) were assembled by SPPS on an automated Symphony^®^ X synthesizer from Gyros Protein Technologies (Tucson, AZ, USA) (Scheme 1). The orthogonal Fmoc/tBu scheme was applied, using a Rink amide MBHA resin as solid support, which was pre-conditioned in dimethylformamide (DMF) for 10 min. The Fmoc protecting group was then removed by treating the resin twice with a solution of 20% piperidine in DMF for 2:30 min (twice), thus releasing the resin-bound reactive amine groups. The *C*-terminal Fmoc-AA-OH was next coupled to the deprotected resin, which was treated twice for 10 min with a cocktail solution containing 100 mM of the Fmoc-AA-OH, 200 mM of HCTU and 400 mM of *N*-methylmorpholine (NMM) in DMF. The Fmoc-protecting group was removed as before to release the amino acid (AA) a-amine group for subsequent coupling of the next Fmoc-AA-OH. Hence, the peptide chain was grown in the C→N direction through alternating coupling and deprotection cycles, performed as above described, until the full sequence was assembled. The peptides were then released from the resin through a 2 h acidolytic cleavage reaction using a cocktail solution containing 87.55% trifluoroacetic acid (TFA), 2.5% triisopropylsilane (TIS), 5% of phenol and 5% of deionized water. The crude peptide thus obtained was purified by preparative high-performance liquid chromatography (HPLC), on a Hitachi-Merck LaPrep Sigma system (VWR International LLC, Carnaxide, Portugal) equipped with an LP3104 UV detector and an LP1200 pump, employing a reverse-phase C18 column (250 × 25 mm^2^ ID and 5 m pore size) and gradient elution using 0.05% TFA in water as solvent A and acetonitrile (ACN) as solvent B. The elution method varied according to the specific peptide and all elutions were completed in 60 min, at a 15 mL/min flow rate. Pure peptide fractions were isolated pooled, and freeze-dried to produce the peptide as a low-density white solid that was stored at −20 °C until further use. Peptide purity was confirmed by analytical HPLC using a Hitachi-Merck LaChrom Elite system equipped with a quaternary pump, a thermostated automated sampler, and a diode array detector; analyses were performed with a reverse-phase C18 column (150 × 4.6 mm^2^ ID and 5 m pore size) at a 1 mL/min flow rate using a 1% to 100% of solvent B in solvent A, for 30 min, with detection at 220 nm. Peptide molecular weight was confirmed by electrospray ionization–ion trap mass spectrometry (ESI-IT MS), and all peptides were accurately quantitated by microvolume spectrophotometry, using the 31 method on a NanoDrop one instrument (Thermo Fisher Scientific, Waltham, MA, USA).

### 2.3. Cell Culture

RAW 264.7 (ATCC TIB-71) murine macrophage-like cells were cultured in high-glucose Dulbecco’s modified Eagle’s medium (DMEM, Sigma-Aldrich) supplemented with 10% fetal bovine serum (FBS, Corning) and 1% penicillin/streptomycin (Corning). Cells were grown under standard culture conditions in a 5% CO_2_ incubator at 37 °C (MCO-170AC incubator; PHC Corporation, Tokyo, Japan), and were sub-cultured every two or three days when it was noticed that the cells had reached around 80% confluence. The assays were performed in cell passages from passages 9 to 21 to avoid phenotypic and genetic instability [33]. Unless otherwise stated, all treatments were carried out with DMEM supplemented with 1% penicillin/streptomycin only (serum-free conditions).

### 2.4. Cell Viability Assay

The cytotoxicity of the peptides towards RAW 264.7 cells was evaluated using the colorimetric 3-(4,5-dimethylthiazol-2-yl)-2,5-diphenyl tetrazolium bromide (MTT) assay. The assay was performed according to Mosmann [34], with a few modifications. Briefly, cells were seeded in a 96-well plate at a density of 2.5 × 10^3^ cells per well and incubated for 24 h (5% CO_2_, 37 °C). After the incubation period, the medium was removed and replaced with different concentrations of the peptides’ aqueous solutions diluted in fresh FBS-free DMEM to the final concentrations of 1–0.005 mg/mL in each well. Following another 24 h incubation period, 10 µL of a 5 mg/mL stock solution of 3-(4,5-Dimethylthiazol-2-yl)-2,5-diphenyltetrazolium bromide (MTT; Alfa Aesar) was added to each well, and the plate was incubated for 3 h (5% CO_2_, 37 °C). Dimethyl sulfoxide (DMSO, Thermo Fisher Scientific) was used to dissolve the formazan crystals formed by the intracellular reduction of MTT. The absorbance was measured at a dual wavelength of 570/690 nm using a Multiskan SkyHigh microplate reader (Thermo Fisher Scientific). The percentage of viable cells was calculated and the concentration of each peptide able to inhibit 50% of cell viability (IC_50_) was determined. The experiments were carried out in three independent assays, and the results are shown as mean ± standard deviation (SD).

### 2.5. DPPH^•^ Radical Scavenging Assay

The free radical-scavenger activity was determined by the DPPH^•^ assay, according to Oliveira et al. [35]. Briefly, in a 96 well-plate, 19.4 µL of aqueous solutions of the peptides at different concentrations (100–0.1 µg/mL) was added per well. Then, 175 µL of an ethanolic solution of 2,2-diphenyl-1-picrylhydrazyl radical (DPPH^•^, Merck) was added to the wells (394 µg/mL—90 µM for final concentration in the experiment). The absorbance was read at 515 nm (Multiskan SkyHigh multiplate reader, Thermo Fisher Scientific) in kinetic mode for 60 min and at room temperature. The percentage of remaining DPPH^•^ was plotted against the peptides’ concentrations and the amount of test compounds necessary to decrease by 50% the initial concentration of DPPH (RIC_50_) was calculated (GraphPad Prism 9.4.0, GraphPad Software). Quercetin (100–0.1 µg/mL in ethanol) was used as a positive control.

The DPPH^•^ radical scavenging activity determines the ability of an antiradical compound to neutralize DPPH^•^ radical to its reduced form (DPPH-H) and is usually expressed as the concentration of the compound required to reduce the DPPH^•^ radical by 50% (IC₅₀). As the molecular weights varied considerably amongst the synthetized peptides, we opted to determine the peptides’ Relative Concentration to Reduce 50% (RIC_50_) of the initial concentration of the DPPH^•^ radical, as recommended by De Menezes et al. [36] to eliminate the molecular weight factor. Moreover, quercetin, a well-known antioxidant, was used as a positive control.

### 2.6. FRAP Assay

The antioxidant power of a given compound is not limited to its radical-scavenging activity. In fact, the ferric reducing antioxidant power (FRAP) assay, is a well-established test to measure a compound’s ability to act as a reductant in a colorimetric redox reaction, where ferric (Fe^3+^) ions are reduced to the ferrous (Fe^2+^) state in the acidic medium [37].

The ferric-reducing antioxidant power (FRAP) was determined using a FRAP kit (Sigma-Aldrich) with minor modifications to the manufacturer’s instructions. Briefly, in a 96-well plate, 5 µL of peptides’ aqueous solutions at different concentrations (50–0.05 µg/mL) was added per well. Then, 95 µL of the reaction cocktail (FRAP assay buffer, FeCl_3_ solution, and FRAP probe) was added to each well. The absorbance was measured at 594 nm in kinetic mode (Multiskan SkyHigh, Thermo Fisher Scientific) for 60 min, at 37 °C. A ferrous standard curve was plotted with Ferrous ammonium sulfate at 0, 4, 8, 12, 16, and 20 nmol/well, and the absorbances of each sample were compared to the standard curve. Quercetin (100–0.1 µg/mL in ethanol) was used as a positive control. All measurements were performed in three independent experiments. Data are shown as mean ± SD.

### 2.7. ABTS^•+^ Radical Scavenging Assay

The ability of compounds to scavenge free radicals in the intracellular milieu is an important factor in determining their antioxidant potential. As such, the ability of the LF-derived peptides under study to scavenge ABTS^•+^ radical-cations was evaluated intracellularly in LPS-activated RAW 264.7 cells, using the total antioxidant status (TAS) assay. This colorimetric method settles on the bright green color of stable ABTS^•+^ radical cations, which fade out in the presence of radical scavenging agents [38,39].

The total antioxidant status (TAS) kit (Randox Laboratories) was used to assess the peptides’ capacity to scavenge the 2,2′-azino-bis(3-ethylbenzothiazoline-6-sulfonic acid) diammonium salt radical cation (ABTS^•+^). The manufacturer’s instructions were adapted to be used in RAW 264.7 cells. These were seeded in a 96-well plate at a density of 7 × 10^4^ cells per well and incubated for 24 h (5% CO_2_, 37 °C). Afterwards, the medium was removed and replaced with the peptides’ aqueous solutions diluted in fresh FBS-free medium to the final concentrations of 50–3.12 µg/mL in each well. Simultaneously, the cells were stimulated with LPS (1 µg/mL). After incubation, 2 µL of the supernatant of each treatment was transferred to a new 96-well plate. Then, 100 µL of the chromogen mixture (metmyoglobin and ABTS) was added and the plate was incubated at 37 °C for 10 min. The absorbance A1 was measured at 600 nm and 37 °C using a microplate reader (Multiskan SkyHigh, Thermo Fisher Scientific). Then, 20 µL hydrogen peroxide was added and absorbance A2 was measured after 3 min, in similar conditions. After subtracting A1 values from the A2 ones, the percentage of radical-cation ABTS^•+^ scavenging activity was calculated. Quercetin (10 µg/mL in ethanol) was used as a positive control. The experiments were carried out in three independent assays and the results are shown as mean ± SD.

### 2.8. Intracellular Determination of Superoxide Production

Considering the link between oxidative stress and inflammation, both the antioxidant and the anti-inflammatory potential of the selected peptides was determined in cellulo using macrophage-like cells (RAW 264.7 cell line) stimulated with lypopolysaccharides (LPSs) from *Escherichia coli*. LPSs are a fundamental component of the outer membrane of Gram-negative bacteria [40], which act as endotoxins hence their use to induce oxidative stress and inflammatory responses in several in cellulo and in vivo studies. In other words, RAW 264.7 cells are activated by foreign stimuli like, e.g., exposure to LPSs. This activation is initiated by the binding of LPSs to cell membrane receptors, which triggers signaling pathways that will result in the generation of proinflammatory mediators (e.g., nitric oxide) and cytokines such as interleukin (IL)-6, IL-8, tumor necrosis factor-alpha (TNF)-α [41,42]. Since proinflammatory mediators are held in several metabolic pathways, including the production of reactive oxygen species (ROS), e.g., the radical-anion superoxide (O_2_^•−^), LPS exposure also leads to oxidative stress [40,43]. Proinflammatory mediators promote the formation of reactive species, which sets off an intracellular signaling cascade that, in turn, increases the expression of proinflammatory genes [44].

The potential inhibitory effect of the peptides on superoxide radical (O_2_^−^) by RAW 264.7 cells activated by LPS (1 µg/mL) was evaluated according to Mitra et al. [38] with some modifications. To this end, peptide solutions at different concentrations (50–3.12 µg/mL—serial dilution) in an FBS-free medium were used. Briefly, 7 × 104 RAW 264.7 cells per well were seeded in a 96-well plate and incubated for 24 h (5% CO_2_, 37 °C). Following the incubation period, the medium was removed and replaced with different concentrations of the peptides. Simultaneously, the cells were activated with LPS (1 µg/mL) and incubated for 2 h (5% CO_2_, 37 °C). Then, the supernatant was removed and 50 µL of nitroblue tetrazolium (NBT, TCI Chemicals^®^) in methanol (1 mg/mL) was added to each well. After an incubation period of 2 h, the cells were washed with 200 µL of absolute methanol (Chem-Lab^®^) and dried at room temperature. The formazan crystals were dissolved in 120 µL of aqueous potassium hydroxide (KOH) (2 M) and 140 µL of DMSO. The absorbance was measured in a microplate reader at a dual wavelength of 620/700 nm. Quercetin (10 µg/mL) was used as positive control. The experiments were performed in three independent assays and the results were expressed as a percentage of O_2_^-^ production.

### 2.9. Griess Assay

The Griess assay is a classical indirect method to measure the production of nitric oxide through the quantification of nitrite anions [45]. As previously mentioned, NO is an inflammatory biomarker that can be produced when LPS-activated macrophages produce proinflammatory mediators and cytokines [41], which in turn elicit the production of reactive species like ROS and the expression/activity of relevant enzymes, including the nitric oxide synthase (NOS) [38].

The Nitrite Assay Kit (Griess Reagent, Sigma-Aldrich) was used to determine the influence of peptide treatments on nitric oxide production by RAW 264.7 cells activated by LPS (1 µg/mL). To this end, peptide solutions at different concentrations (50–3.12 µg/mL—serial dilution) in an FBS-free medium were used. Briefly, 7 × 10^4^ RAW 264.7 cells per well were seeded in a 96-well plate and incubated for 24 h (5% CO_2_, 37 °C). On the next day, the medium was removed and replaced with different concentrations of the peptides. After another 24 h incubation period (5% CO_2_, 37 °C), 50 µL of culture supernatant was transferred to a 96-well plate. Then, 5 µL of each Griess I and Griess II reagents were added, followed by the addition of 40 µL of the nitrite assay buffer. The absorbance was measured at 540 nm using a microplate reader (Multiskan SkyHigh, Thermo Scientific) A nitrite standard curve was plotted with nitrite standards at 0, 2, 4, 8, and 10 nmol/well, and the absorbances of each sample were compared to the standard curve. Quercetin (10 µg/mL in ethanol) was used as a positive control. The results of three independent experiments are shown as mean ± SD.

### 2.10. ACE-1 Inhibition Assay

The angiotensin-converting enzyme 1 (ACE-1) plays an important role in the renin–angiotensin–aldosterone system (RAAS) that converts angiotensin 1 into angiotensin 2 and degrades bradykinin, while controlling arterial blood pressure as well as salt and water balance [29,46]. Therefore, ACE-1 inhibition activity is often used as one way to measure the anti-hypertensive potential of compounds. Therefore, the ability of the LF-derived peptides to inhibit ACE-1 was evaluated. We opted to include in this assay all LF-derived peptides produced, as ACE-1 inhibition ability is not directly related to antioxidant or anti-inflammatory ability. Moreover, captopril, a potent pharmacological ACE-1 inhibitor, was used as positive control.

The activity of the ACE-1 enzyme was measured using a colorimetric ACE-1 Inhibitor Screening Kit (Abcam) with minor modifications to the manufacturer’s instructions. Briefly, in a 96-well plate, 12.5 µL of peptide’s aqueous solutions at different concentrations (100–0.1 µg/mL) were added per well. Then, 20 µL of diluted ACE-1 enzyme solution was added to each well. The total volume of each well was adjusted to 100 µL and the plate was incubated at 37 °C for 15 min. After the incubation period, 25 µL of the reaction mixture (substrate mixture and ACE-1 assay buffer) were added to the samples, and the absorbance was read in kinetic mode at 345 nm for 60 min at 37 °C (Multiskan SkyHigh, Thermo Scientific). Captopril (22 ng/mL) was used as the positive control. The reaction inhibition percentage was calculated and the IC_50_ was determined (GraphPad Prism 9.4.0, GraphPad Software). The experiments were carried out in three independent assays and the results are expressed as mean ± SD.

### 2.11. Statistical Analysis

The statistical analyses were performed using GraphPad Prism^®^ version 9.4.0 for Windows (GraphPad Software). At least three independent assays (n = 3) with a minimum of two replicates were performed and the results are expressed as mean ± SD. One-way *ANOVA* followed by Dunnett’s post hoc test was used for analysis of variance and to compare differences observed between the treatments and/or different samples.

## 3. Results

### 3.1. In Vitro Characterization of LF-Derived Peptides Antioxidant Activity and Cytotoxicity

To explore the possible antioxidant activity of LF-derived bioactive peptides, their radical scavenging activity and reducing ability were evaluated in vitro by the DPPH^•^ radical scavenging activity and ferric reducing antioxidant power (FRAP) assays, respectively.

LF-derived bioactive peptides exhibited variable DPPH^•^ radical scavenging activities (Table 2), ranging from 1.74 ± 0.60 to 6.14 ± 0.56 (RIC_50_ mol/mol). bLF-17-31 presents the highest radical-scavenging activity, with an RIC_50_ value of 1.74 ± 0.6 mol/mol DPPH^•^, slightly higher than that of quercetin (0.09 ± 0.02 mol/mol DPPH^•^).

Therefore, 0.05, 0.5, 5, and 50 µg/mL peptides’ aqueous solutions were submitted to FRAP assay. As shown in Table 3, bLF-1-11 and bLF-17-31 stood out again, alongside rhLF-1-11, as the peptides exhibiting the highest reducing power with, respectively, 0.46 ± 0.03 and 0.45 ± 0.02 mM Fe^2+^ equivalents at a concentration of 50 µg/mL. The highly potent antioxidant quercetin displayed a reducing power of 16.09 ± 0.87 mM Fe^2+^ equivalents at the same concentration.

**Figure 1:**
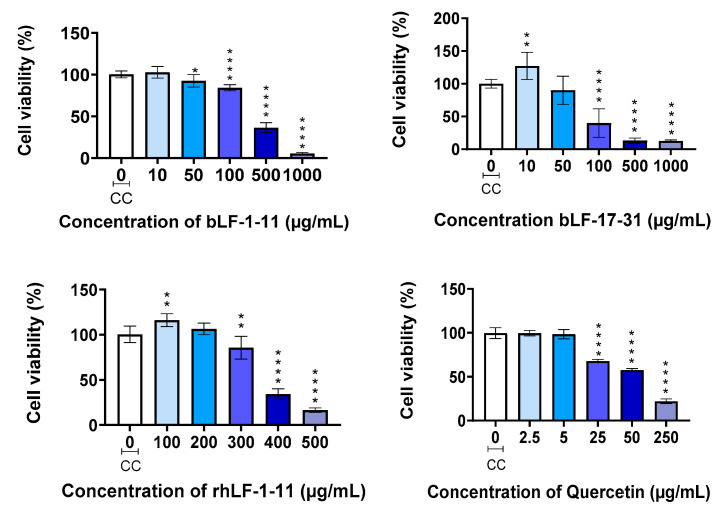
Cell viability determined by the MTT assay: The results are expressed as the mean ± SD of three independent experiments (n = 3). * *p* < 0.05; ** *p* < 0.01; **** *p* < 0.0001 vs. Cell control (CC).

Based on their antioxidant activities, the most promising peptides, bLF-1-11, bLF-17-31, and rhLF-1-11, were selected for further evaluation of both antioxidant and anti-inflammatory properties in cellulo. Prior to advancing with these studies, the non-cytotoxic dosages of the peptides towards RAW 264.7 macrophage-like cell line were evaluated. To this end, cell viability determination by the MTT assay was employed, and quercetin was included as a control (Figure 1).

In general, the three peptides showed low toxicity in RAW 264.7 cells, with IC_50_ values of 135 ± 6, 290 ± 5, and 355 ± 12 µg/mL for LF-17-31, bLF-1-11, and rhLF-1-11, respectively. rhLF-1-11 is the less cytotoxic peptide of the set. According to these results, the optimal concentrations of the peptides should range from 50 to 3.12 µg/mL (serial dilutions) for the antioxidant and anti-inflammatory experiments to be performed using the RAW 264.7 cell line.

Because of its well-known antioxidant and anti-inflammatory properties [47], the flavonoid quercetin was also chosen as a positive control for the future in cellulo assays. Therefore, its toxicity to RAW 264.7 macrophage-like cells was also assessed. Quercetin exhibited a slightly higher toxicity than the test peptides against the target cells, with an IC_50_ value of 65 ± 2 µg/mL. Hence, a concentration of 10 µg/mL of quercetin was standardized as the positive control for the ensuing antioxidant and anti-inflammatory activity assays.

### 3.2. LF-Derived Peptides in Cellulo Assays

#### 3.2.1. Results for Intracellular Determination of Superoxide Production

Therefore, the nitroblue tetrazolium (NBT) assay was used to measure the intracellular production of the superoxide radical-anion, O_2_^•−^, a well-known major player in oxidative stress [44]. The intracellular inflammatory/oxidative stress increases the production of O_2_^•−^ in cells, which leads to a greater reduction in NBT that results in the formation of green-blue formazan crystals [38]. This reaction can therefore be used to evaluate the antioxidant potential of the peptides studied in this work.

**Figure 2:**
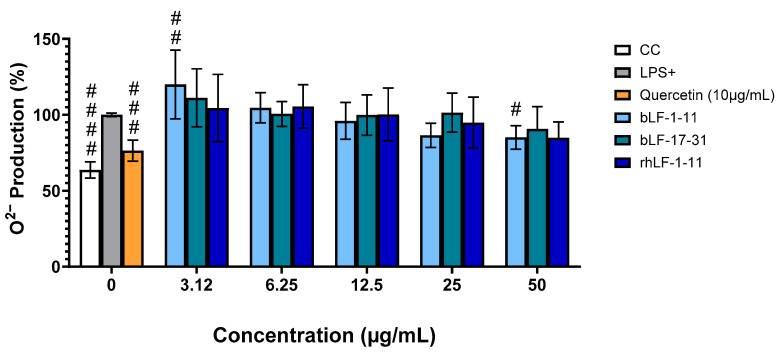
Effect of the peptides on O_2_^•−^ production in LPS-stimulated macrophages. RAW 264.7 macrophage-like cells were treated with different concentrations of the peptides and stimulated with LPS at 1 μg/mL. The results were expressed as mean ± SD of three independent experiments. Significant when compared to LPS induced cells # *p* < 0.05; ## *p* < 0.01, ### *p* < 0.001; #### *p* < 0.0001. CC: cell control; LPS+: LPS-stimulated macrophages; Q: quercetin at 10 μg/mL.

As shown in Figure 2, control cells (CC), i.e., non-stimulated by LPS, showed low production of superoxide, whereas this radical anion was significantly produced in LPS-stimulated cells (LPS +). Interestingly, treatment of LPS-stimulated macrophages with the test peptides apparently exerts a dose-dependent response, suggesting the peptides’ ability to inhibit the production of O_2_^•−^ at high concentrations. This is in line with the IC_50_ values estimated namely, 133 ± 13 µg/mL, 291 ± 36 µg/mL, and 183 ± 25 µg/mL for bLF-1-11, bLF-17-31 and rhLF-1-11, respectively. Considering the cytotoxic concentrations for these peptides (see Section 3.1), peptide rhLF-1-11, derived from the recombinant human LF protein, could significantly inhibit O_2_^•−^ production in LPS-stimulated cells at a concentration much below its cytotoxic concentration. The positive control quercetin, at 10 mg/mL, reduced O_2_^•−^ production by about 25% compared to the control (LPS +).

#### 3.2.2. Results for the ABTS^•+^ Radical-Cation Scavenging Assay

**Figure 3:**
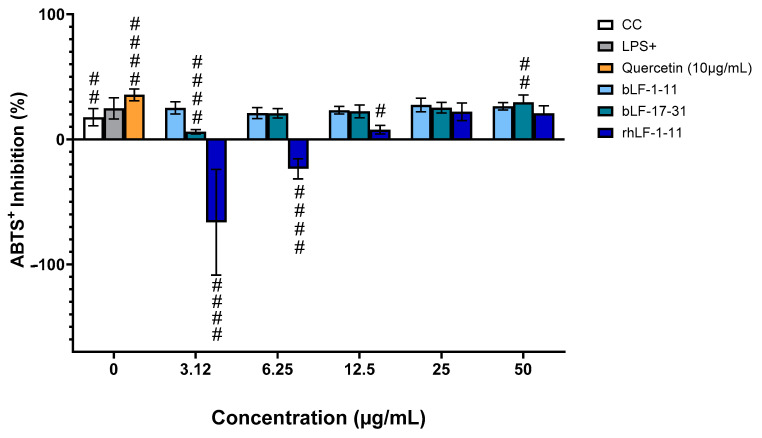
Effect of the peptides on ABTS^•+^ scavenging activity in LPS-stimulated macrophage-like cells. These were treated with various concentrations of the peptide and stimulated by LPS at 1 μg/mL. After a 24 h incubation period, the supernatant was collected and the percentage of ABTS^•+^ scavenging activity was determined using the TAS kit. Results were expressed as mean ± SD of three independent experiments, # *p* < 0.05; ## *p* < 0.01, #### *p* < 0.0001 compared to LPS induced cells.

**Figure 4:**
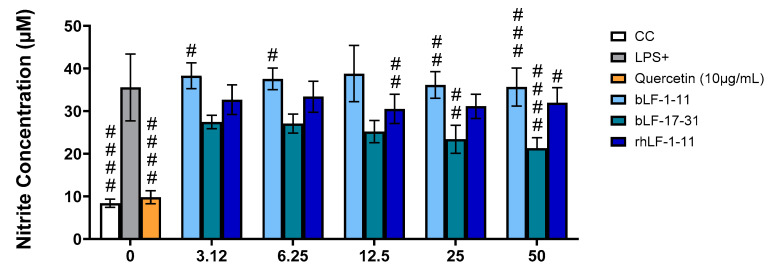
Effect of the peptides on NO production in LPS-stimulated macrophages. Results were expressed as mean ± SD of three independent experiments, # *p* < 0.05; ## *p* < 0.01, ### *p* < 0.001; #### *p* < 0.0001. CC: cell control; LPS+: LPS-stimulated macrophages; Q: quercetin at 10 μg/mL.

The results for the ABTS•+ Radical-Cation Scavenging Assayis presented in Figure 3 and the TAS of the LF-derived peptides is presented in Figure 4. Consistent results could only be obtained for peptide bLF-17-31 (Figure 3), which showed scavenging activity with an IC_50_ of 105.31 ± 18.92 µg/mL.. Both this fact and the unexpected observation that LPS-activated (LPS +) cells scavenged ABTS^•+^ more effectively than non-activated ones (CC) remain to be elucidated.

#### 3.2.3. Results for Griess Assay

As such, we evaluated the influence of the LF-derived peptides under study on NO generation in LPS-stimulated RAW 264.7 cells by the Griess method. LPS-activated cells (LPS +) showed much higher levels of NO than non-activated ones (CC), whereas quercetin was able to significantly reduce NO levels in LPS-activated cells at 10 mg/mL (Figure 4). The test peptides showed a much lower ability to inhibit NO formation, although all of them seemingly exert some level of inhibition. As seen in Table 4 peptide bLF-17-31 showed the lowest IC_50_ with 44.07 µg/mL.

Comparing the percentage of inhibition of the three peptides at the highest concentration tested (50 mg/mL), bLF-17-30 is confirmed as the most active of the set, with 22.5% reduction in NO, compared to 18.5 and 9.9% for bLF1-11 and rhLF-1-11, respectively.

### 3.3. Results for the ACE-1 Inhibition Assay

As shown by IC_50_ values listed in Table 5, peptides aLF-17-31, bLF-17-31, and nhLF-268–284 all displayed comparable activity, with the latter being apparently the strongest inhibitor of the set. However, none of the peptides were as strong as the reference drug captopril in inhibiting ACE-1 activity.

## 4. Discussion

The results of the in vitro radical scavenging activity and ferric reducing power for the LF-derived peptides herein studied are in line with the antioxidant properties that have been ascribed to diverse LF proteins, probably due to their strong affinity for iron. This affinity could be related to the fact these proteins are responsible for the regulation and transportation of iron within the body while protecting it against the oxidative effects of iron [48]. Through iron sequestration, LFs prevent the undesired effects of oxidative stress by managing the physiological equilibrium of iron-induced ROS, therefore contributing to homeostasis [49,50].

The antioxidant activity of milk and derived LFs, even from minor dairy species like the donkey, have been well recognized [51,52,53]. The same applies to LFs from other mammalian species, including humans: in 1999, Belizi et al. described a high radical-scavenging for human (*Homo sapiens*) LF [54]; although having slight structural differences, both native human LF (nhLF) and recombinant human (rhLF), obtained using different expression systems including transgenic animals and plants [55], were able to reduce ROS levels in pre-treated cells with no significant statistical difference [56]. In 2022, Narmuratova et al. compared the DPPH^•^ radical scavenging capacity of purified equine lactoferrin (eLF) with its bovine orthologue (bLF) [57]; these authors found that both LFs showed a DPPH^•^ radical scavenging capacity of approximately 33% at a concentration of 500 µg/mL, suggesting that such activity is species-independent. In an earlier study, Habib et al. [55] determined the DPPH^•^ radical scavenging capacity of isolated camel lactoferrin (cLF) and obtained a 2.77% of DPPH^•^ inhibition at 100 µg/mL; the same authors also determined a ferric reducing power of 0.317 mM Fe^2+^ at a concentration of 100 µg/mL for cLF [58]. LF-derived peptides herein studied were all able to scavenge DPPH^•^ radicals, hence reflecting the same ability formerly reported for LF proteins of diverse origins. Relevantly, the most active peptides bLF-17-31, bLF-1-11, and rhLF-1-11 displayed higher DPPH^•^ radical scavenging capacity than those previously reported for eLF and cLF [54,55,57,58]. In other words, our findings suggest that these three peptides could have better antioxidant activity than those LFs. Likewise, the ferric reducing power of all these peptides was higher than that reported for cLF [58], which contributes to the advancement of bioactive LF-derived peptides instead of whole proteins, given the advantages of short peptides as therapeutically relevant entities, such as easier and more cost-effective production; higher resistance to denaturation; and lower propensity to trigger immune (i.e., allergic) reactions [59,60]. In this case, the concentrations of the peptides do not have any effect on ferric-reducing power. Nevertheless, the values obtained showed better antioxidant properties for our peptides than the ones described in the literature above [58]

Previous studies highlighted the anti-inflammatory activity of LFs [61]. Likewise, LF hydrolysates were reported to produce bioactive peptides with anti-inflammatory properties in human cartilage and synovial cells, suggesting a potential benefit in arthritis treatment [62]. Moreover, LFs were found to reduce the effects of LPS by direct and indirect mechanisms, including their ability to neutralize LPS and to prevent LPS interactions with cell membrane receptors [63]. Consistently, we observed anti-inflammatory effects for the peptides herein studied, with those derived from bovine LFs, namely, bLF-1-11,bLF-17-31, and rhLF1-1, showing the best results; these were actually superior to those previously reported by Habib et al. for cLF, which inhibited NO production by about 5% at a concentration of 100 µg/mL [58]. This is consistent with the fact that bLF peptides are known to interact with negatively charged groups present on the surface of immune cells, activating signaling pathways that induce an anti-inflammatory reaction [64].

Regarding the potential anti-hypertensive potential of LFs and derived peptides, several reports highlighted the ACE-1 inhibitory effects of LF-derived peptides: in 2006, Lee et al. identified the bLF-derived peptide LRPVAA as effective in inhibiting ACE-1 in vitro and in reducing blood pressure in spontaneously hypertensive rats [65]; very recently, Oussaief et al. reported ACE-1 inhibitory activity in camel milk, and further found that cLF hydrolysate displayed 89% ACE-1 inhibitory activity of at a concentration of 10 mg/mL with an IC_50_ value of 4.02 mg/mL [66]. Earlier in 2006, Centeno et al. also reported a significant ACE-1 inhibitory activity for peptide bLF-17-31 in vitro and ex vivo [29]; these authors found an IC_50_ of 25.5 ± 4.5 µM for this peptide, which is in the same order of magnitude as the value herein reported.

Our findings contribute to consolidating the value of milk-related peptides as biologically active natural products with many prospective applications in health and well-being. This is reinforced by the fact that many such peptides have also antimicrobial activity [23,24,25]. Specifically, LF-derived peptides hold great promise as candidate nutraceuticals for the future development of functional foods. This motivates additional studies in such regard, not only to clarify some of the observations made in this work, but also to explore other related peptides, and to investigate relevant aspects such as their chemical and biochemical stability, as well as in vivo pharmacokinetics and pharmacodynamics of the best candidates’ nutraceuticals. Another important studies that have to be made is finding more sustainable and environmentally friendly peptide synthesis.

## 5. Conclusions

We produced non-toxic MDBPs with antioxidant, anti-inflammatory, and antihypertensive activities, with those derived from bovine and human LFs showing the most promising profiles. Although the activities displayed by the peptides were generally not as expressive as those obtained for the positive controls, quercetin, and captopril, their natural (hence, biodegradable and eco-friendly) origin, alongside their expected high accessibility as they can be obtained by hydrolysis of whey proteins, offers high prospects for their future development as nutraceutical agents. Furthermore, this work contributes to consolidating the role of LFs and derived peptides as extremely versatile biomolecules with a wide range of biological activities.

## Figures and Tables

**Table 1:** List of lactoferrin-derived peptides under study.

Peptide	Origin	Amino Acid Sequence	Molecular Weight (g.mol^−1^)	Representative References
aLF-17-31	*Equus africanus asinus*	AKCAKFQRNMKKVRG-NH2	1762.99	[27]
bLF-1-11	*Bos taurus*	APRKNVRWCTI-NH2	1341.74	[28]
bLF-17-31	*Bos taurus*	FKCRRWQWRMKKLGA-NH2	1992.09	[29]
bLF-268-284	*Bos taurus*	WKLLSKAQEKFGKNKSR-NH2	2046.18	[30]
nhLF-268-284	*Homo sapiens*	WNLLRQAQEKFGKDKSP-NH2	2043.10	[31]
rhLF-1-11	*Recombinant human LF*	GRRRRSVQWCA-NH2	1372.74	[32]

**Table 2:** DPPH radical scavenging activity of the synthetized peptides.

Compound	DPPH Radical Scavenging Activity (RIC50 mol/mol DPPH)
aLF-17-31	6.14 ± 0.56 ****
bLF-1-11	2.78 ± 0.83 **
bLF-17-31	1.74 ± 0.60
bLF-268-284	5.16 ± 0.49 ****
nhLF-268-284	2.96 ± 0.91 **
rhLF-1-11	2.47 ± 0.20 *
Quercetin	0.09 ± 0.02

Results are shown as mean ± SD (n = 3). * *p* < 0.05 vs. quercetin; ** *p* < 0.01 vs. quercetin; **** *p* < 0.0001 vs. quercetin.

**Table 3:** Ferric reducing activity of the LF-derived peptides.

Compound	FRAP (mM Ferrous Equivalents)			
	0.05 µg/mL	0.5 µg/mL	5 µg/mL	50 µg/mL
aLF-17-31	0.16 ± 0.02	0.17 ± 0.04	0.18 ± 0.04	0.19 ± 0.03
bLF-1-11	0.20 ± 0.07	0.20 ± 0.07	0.23 ± 0.07	0.46 ± 0.03
bLF-17-31	0.18 ± 0.04	0.19 ± 0.05	0.24 ± 0.06	0.35 ± 0.02
bLF-268-284	0.17 ± 0.04	0.18 ± 0.04	0.17 ± 0.02	0.29 ± 0.11
nhLF-268-284	0.18 ± 0.04	0.17 ± 0.03	0.19 ± 0.05	0.24 ± 0.02
rhLF-1-11	0.20 ± 0.05	0.21 ± 0.07	0.26 ± 0.07	0.45 ± 0.02
Quercetin	0.85 ± 0.25	1.40 ± 0.23	8.02 ± 0.57	16.09 ± 0.87

Results are shown as mean ± SD (n = 3) and *p* < 0.0001 vs. quercetin.

**Table 4:** Inhibitory concentration (IC50) of NO in µg/mL in the Gries Assay (inhibition of nitric oxide production).

Compound	IC_50_ /µg/mL
bLF-1-11	78.32 ± 8.31
bLF-17-31	44.07 ± 4.67
rhLF-1-11	108.58 ± 23.78

The experiments were performed in three independent replicates.

**Table 5:** ACE-1 inhibitory activity of test compounds.

Compound	IC_50_ /µg/mL
aLF-17-31	55 ± 8
bLF-1-11	<100
bLF-17-31	63 ± 5
bLF-268-284	>100
nhLF-268-284	52.3 ± 0.8
rhLF-1-11	79 ± 9
Captopril	0.0070 ± 0.0004

The experiments were performed in three independent replicates. All results were considered significant when *p* < 0.0001 compared to the same concentration of Captoril.

## Data Availability

The original contributions presented in the study are included in the article, further inquiries can be directed to the corresponding author.

## Abbreviations

The following abbreviations are used in this manuscript:

AA	Amino acid
ABTS•+	2,2′-azino-bis(3-ethylbenzothiazoline-6-sulfonic acid) diammonium salt radical cation
ACN	Acetonitrile
bLF	Bovin Lactoferrins
CC	Control cells
cLF	Camel lactoferrin
DMEM	Dulbecco’s modified Eagle’s medium
DMF	Dimethylformamide
DPPH	2,2-diphenyl-1-picrylhydrazyl radical
eLF	equine lactoferrin
ESI-IT MS	electrospray ionization-ion trap mass spectrometry
FBPs	Food-derived bioactive peptides
FBS	Fetal bovine serum
FRAP	ferric reducing antioxidant power
hLF	Human Lactoferrins
HPLC	High-performance liquid chromatography
IC50	inhibit 50% of cell viability
LPS	Lipopolysaccharide
MDBPs	Milk-derived bioactive peptides
MTT	3-(4,5-dimethylthiazol-2-yl)-2,5-diphenyl tetrazolium bromide
NBT	Nitroblue tetrazolium
nhLF	Native human Lactoferrin
NMM	N-methylmorpholine
RAAS	Role in the renin–angiotensin–aldosterone system
rhLF	Recombinant human Lactoferrin
RIC50	Relative Concentration To Reduce 50%
SD	Standard deviation
SPPS	Solid-phase peptide synthesis
TAS	Total antioxidant status
TFA	Trifluoroacetic acid
TIS	Triisopropylsilane
